# Economic, social and mental health impacts of an economic intervention for female sexual violence survivors in Eastern Democratic Republic of Congo

**DOI:** 10.1017/gmh.2016.13

**Published:** 2016-06-06

**Authors:** J. Bass, S. Murray, G. Cole, P. Bolton, C. Poulton, K. Robinette, J. Seban, K. Falb, J. Annan

**Affiliations:** 1 Department of Mental Health, Johns Hopkins Bloomberg School of Public Health, Baltimore, Maryland, USA; 2 Ministry of Human Services, Government of Alberta, Edmonton, Alberta, Canada; 3 Department of International Health and Center for Refugee and Disaster Response, Johns Hopkins Bloomberg School of Public Health, Baltimore, Maryland, USA; 4 International Rescue Committee, Brussels, Belgium; 5 International Rescue Committee, Kinshasa, Democratic Republic of Congo; 6 Laboratory for Interdisciplinary Evaluation of Public Policies, Sciences Po University, Paris, France; 7 International Rescue Committee, New York, New York, USA

**Keywords:** Economic intervention, interventions, LMIC, mental health, sexual violence

## Abstract

**Background.:**

Conflict-affected communities face poverty and mental health problems, with sexual violence survivors at high risk for both given their trauma history and potential for exclusion from economic opportunity. To address these problems, we conducted a randomized controlled trial of a group-based economic intervention, Village Savings and Loans Associations (VSLA), for female sexual violence survivors in the Democratic Republic of Congo.

**Methods.:**

In March 2011, 66 VSLA groups, with 301 study participants, were randomized to the VSLA program or a wait-control condition. Data were collected prior to randomization, at 2-months post-program in June 2012, and 8-months later for VSLA participants only. Outcome data included measures of economic and social functioning and mental health severity. VSLA program effect was derived by comparing intervention and control participants' mean changes from baseline to 2-month follow-up.

**Results.:**

At follow-up, VSLA study women reported significantly greater *per capita* food consumption and significantly greater reductions in stigma experiences compared with controls. No other study outcomes were statistically different. At 8-month follow-up, VSLA participants reported a continued increase in *per capita* food consumption, an increase in economic hours worked in the prior 7 days, and an increase in access to social resources.

**Conclusions.:**

While female sexual violence survivors with elevated mental symptoms were successfully integrated into a community-based economic program, the immediate program impact was only seen for food consumption and experience of stigma. Impacts on mental health severity were not realized, suggesting that targeted mental health interventions may be needed to improve psychological well-being.

## Background

The link between poverty and mental health conditions is well established; globally, people living in poverty have higher rates of common mental disorders and individuals with common mental disorders often drift into poverty (Lund *et al.* 2011; Haushofer & Fehr, 2014). Traditional intervention research for people with mental health conditions living in poverty has focused on alleviating the burden of symptoms through psychotherapy and/or psychosocial programming (Lund *et al.* 2011). Research is now expanding examining the impact of economic interventions on mental health symptoms. For example, Fernald *et al*. (2008) found that a loan program increased psychological stress but reduced depressive symptoms among participants compared with controls. More recently, Haushofer & Shapiro (2013) and Ozer *et al.* (2011) found that cash transfer programs reduced depression symptoms when compared with control participants.

Conflict-affected communities often face the dual burden of widespread poverty and mental health problems. One group that is particularly vulnerable is survivors of sexual violence, who are at high risk for mental health problems generally (Resick, 1993; Chivers-Wilson, 2006; Tjaden & Thoennes, 2006; Campbell *et al.* 2009; Chen *et al.* 2010; Booth *et al.* 2011), and in settings affected by conflict (Loncar *et al.* 2006; Roberts *et al.* 2008; Johnson *et al.* 2010; Betancourt *et al.* 2011). Concurrent with the mental health consequences of sexual violence, many female survivors also experience social stigmatization leading to exclusion from economic opportunity and an increased risk of poverty (Kelly *et al.* 2011; Kohli *et al.* 2014).

Sexual violence is a significant problem for women living in the eastern provinces of the Democratic Republic of the Congo (DRC) (Johnson *et al.* 2010), where persistent conflict and insecurity has led to large-scale civilian death and displacement. While rape by armed groups continues, recent reports indicate that perpetrators are both armed actors and civilians, including intimate partners (Duroch *et al.* 2011; Peterman *et al.* 2011; Bartels *et al.* 2013). Survivors have described a range of mental health and psychosocial problems including mood disorders, anxiety, withdrawal, and stigmatization and rejection by family and community (Johnson *et al.* 2010; Kelly *et al.* 2011).

There is limited evidence for interventions that address the complex burdens of mental health issues, psychosocial challenges, and economic hardship among survivors of sexual violence in conflict settings. Group-based economic activities may provide a means of impacting economic outcomes and mental health through providing an environment for social connection and solidarity among group members (Pronyk *et al.* 2008), which may in turn lead to improvements in mental health. Group activities may also create an environment in which participants can experience recovery within a social context characterized by relationships with others (Herman, 1997). In collaboration with the International Rescue Committee (IRC), we tested this hypothesis by conducting a randomized controlled trial of a group-based economic intervention in the South Kivu province of eastern DRC. We investigated the impact of Village Savings and Loans Associations (VSLA) on economic, social, and psychological outcomes among female sexual violence survivors with elevated mental health symptoms and impaired functioning.

## Methods

### Intervention: VSLA groups

VSLAs are an economic program in which individuals join together with people they know and trust to regularly save funds and build capital to generate small loans to members within the group that are paid back with fixed interest. This program model was first formalized by the international non-governmental organization, CARE (http://www.care-international.org/) and has been replicated by other organizations globally. The VSLA program continues for a cycle of 9–12 months at the end of which all loans are repaid and the original savings plus interest is shared back with all group members. More information about the VSLA implementation for this study is available in the online Supplementary Appendix.

We hypothesized that participation in VSLAs would provide women who lacked access to financial services access to savings and loans in the safety of a trusted group. Providing a place to save and access to loans would allow them to invest in small-scale enterprise or economic opportunities that require capital for a return and therefore increase their consumption and assets. Based on the understanding that poverty and lack of access to resources can be major stressors (Haushofer & Fehr, 2014), we hypothesized that improving these outcomes would lead to a reduction in stress and accompanying mental health symptoms. We also hypothesized that for women who had experienced sexual violence, participation in the group-based economic program would improve their ability to care for themselves and contribute to their family's well-being, resulting in improved self-efficacy and a reduction in mental health symptoms. Finally, we hypothesized that women who had experienced sexual violence would improve their social connectedness through being part of the economic group and that this, together with increased economic benefits, would reduce their experience of stigma.

### Site selection

Nine communities in South Kivu were selected for the VSLA program. These communities were selected based on IRC's existing partnerships with local Community-Based Organizations (CBOs). The sites were situated along the three geographic axes of Kabare, Uvira and Walungu, which were in driving distance from the IRC Bukavu office to ensure that IRC staff would have regular and consistent access to study sites. Prior to program implementation, IRC staff held meetings with local authorities and CBO leadership to introduce the VSLA program and the study. Local authorities in all sites gave permission to implement the proposed activities.

### Sample selection

In October 2010, IRC staff worked with CBO management committees to identify potential participants to be screened for study eligibility with a previously validated mental health and functionality assessment, described elsewhere (Bass *et al.* 2013). The CBOs were asked to identify women in their community who had self-reported to them for support with significant functioning problems or mental health symptoms, potentially related to trauma. CBO members were given key talking points and visited all potential participants to provide information about the study, reviewed demographic eligibility criteria (being 18 years of age or older and living in one of the nine study sites) and administered oral consent. All women who provided consent to the CBO member were administered the full baseline questionnaire by an independent interviewer. Study eligibility included reporting personally experiencing or witnessing sexual violence (defined as rape locally), a score of at least 10 on the function assessment (i.e. some dysfunction on at least half of the tasks questions – described in the assessment section below), a score of at least 55 on the mental health assessment (i.e. an average score of 1 for each symptom – described in the assessment section below), and not expressing severe suicidality. If severe suicidality was identified, the interviewer and IRC staff referred the participant for immediate services. Study protocols were reviewed and approved by IRBs at the Johns Hopkins School of Public Health and Kinshasa School of Public Health.

### VSLA group formation and randomization

For this study we introduced a variation to the typical VSLA group formation process, which relies on community members learning about the VSLA program in open presentations and then an invitation going out for people in the community to voluntarily create groups of 15–25 people to participate as a VSLA group. The self-selection process is seen as important for members to trust each other enough to build a saving and loaning community. To retain the self-selection process, we held initial meetings about the VSLA program with study-eligible women prior to opening up the program to all women in the community. This adaption allowed the program to target a specific population of survivors of sexual violence with elevated mental health problems, while continuing to respect the self-selection principle that is essential to VSLA programming.

During a first introductory meeting with study eligible women, IRC staff explained the principles of VSLA and obtained informed consent. Women were then asked to return to a second meeting with 2–3 friends from their community who were interested in learning about VSLAs; these 2–3 friends did not have to be research eligible women. At this second meeting, attending women were asked to form groups of women for the VSLA program from the communities in which they lived.

Two community mobilization meetings were organized per site. A total of 113 VSLA group applications were collected across the nine sites. The group applications, which included each member's name and age, were reviewed by IRC staff to identify research-eligible women. Of the 459, research-eligible women identified during the baseline assessment, 305 (66%) self-selected into 70 VSLA groups. Women who did not self-select into any of the groups (*n* = 154) were not different on any of the demographic, mental health and functioning outcomes from those who did self-select into groups, though they did report, on average, 5 fewer hours of total economic work per week compared with the women who self-selected into the VSLA groups (*p* = 0.02). The number of study eligible women who self-selected into VSLA groups ranged from 1 to 12 per group. Four VSLA groups with only one study eligible woman were not included in the study, resulting in 66 VSLA groups with 301 study women available for randomization in March 2011. The 66 groups were randomized into immediate start (intervention; *n* = 33 groups) and delayed start (control; *n* = 33 groups). The control groups did not receive VSLA training until year 2, when follow-up data collection was completed. The intervention period ran from April 2011 to April 2012. One VSLA group was delayed during the training phase and completed in June 2012.

### Measures

For the impact evaluation, all outcomes were assessed using a single questionnaire administered twice to VSLA and control participants. VSLA participants completed a third assessment to identify long-term trends after the program was complete. The questionnaire was translated into three local languages (Mashi, Swahili, and Kifuliru) through the use of qualitative data for the mental health and functioning questions and through collaboration with multi-lingual local staff for the economic, demographic, and social questions. Pilot testing allowed for refinement of the questions to ensure local comprehension and interpretability.

#### Economic outcomes

To measure economic functioning and living standard, two constructs were used: (1) women's participation in the labor market and (2) household assets and food consumption. For participation in the labor market, work was defined as all activities with direct material benefits for the woman and her household. This included paid economic work: wage employment (either in cash or in kind), self-employment (e.g. small business, commerce of agricultural products); and unpaid economic work: in family-run business and cultivation of household's fields. Indicators of labor market participation were measured in the 7 days preceding the assessment, were total hours of economic work (paid and unpaid) and total hours of specifically paid economic work. Extreme outliers at any assessment (more than 90 h per week) for paid hours (*n* = 4), unpaid economic hours (*n* = 4), and total hours of economic work (*n* = 36) were replaced with the sample median.

As an indicator of an asset that might see short-term change, we tracked the number of breeding animals the woman reported. For food consumption, we calculated the monetary value of the household's food consumption during the 7 days preceding the interview. Following common practice, we asked for purchases and own consumption of an itemized list of foodstuff commonly consumed in the region. Own-consumption (that is, consumption of food the woman produced/grew herself) was converted to monetary values using current market prices. The total monetary value of all foodstuffs was divided by household size to arrive at per person consumption expenditures. Responses of ‘I don't know’ for food consumption information were imputed. We replaced extreme outliers at all time points (top 2–3% of the sample) with median values (*n* = 17).

VSLA monitoring data and additional economic outcomes were also collected. As part of the monitoring system, each group kept a log of the amount of savings brought in each week and at the end of the cycle, the amount of funds paid as share-out to all group participants. These data were collected for the groups as a whole, not differentiating between trial and non-trial participants. Additional economic outcomes are presented in the online Supplementary Appendix as results are similar to the main outcomes described above: any paid work in the past 7 days (yes/no), total hours of unpaid work in the past 7 days (unpaid economic work and domestic work), and total hours of work regardless of paid or unpaid in past 7 days. In addition, a full asset index was calculated and evaluated.

#### Social functioning and stigma outcomes

To understand changes in social functioning, we investigated access to social resources, participation in community groups, and feelings of stigma. Access to social resources was assessed using two scenario questions: (1) if you suddenly needed a small amount of money, for example like enough to pay for your household for 1 week, how many people could you turn to who would be willing to provide this money, and (2) if you were suddenly faced with a long-term emergency, such as a family death or harvest failure, how many people could you turn to who would be willing to assist you? The average number of people each respondent could access across the two questions was used as an indicator of access to social resources. Due to the right skewed distribution of this variable, we replaced extreme outliers at any assessment with median values (*n* = 17).

Group participation was assessed using a combination of membership in any of nine community groups (i.e. farming or production, folkloric dance, religious or spiritual, cultural, health, solidarity, education/literacy, community-based organization/non-governmental organization, women's group) and level of participation (responses ranging from 0 = not at all, 1 = sometimes, but not often, 2 = most of the meetings, 3 = every meeting) using methods described elsewhere (Hall *et al.* 2014). For each of the nine groups, non-membership was coded 0, and then membership with participation was coded as follows: no participation = 0.25, sometimes but not often participation = 0.50, participation in most meetings = 0.75, and participation in all meetings = 1. Each participant received a group participation score ranging from 0 to 9, with higher scores indicating more participation.

Stigma was assessed using an 8-item scale containing items related to perceived and internalized stigma generated from an initial qualitative study of problems faced by sexual violence survivors in eastern DRC. The scale's development process, psychometrics, and validity are presented elsewhere (Murray *et al.* 2015). For each item, respondents indicated how often they experienced it in the past 2 weeks on a Likert scale of 0 (not at all) to 3 (a lot). Responses to scale items were averaged with participants' scores ranging from 0 to 3; higher scores indicate greater stigma.

Additional social functioning variables are presented in the online Supplementary Appendix including a social coping scale made up of three coping items: talk about your problems with friends or family; talk about your problems with other women who have experienced similar traumas; and spend time with others and 2 subscales from the daily functioning measure focusing on interacting with members of the community and family members. Lower scores indicate less difficulty functioning.

#### Mental health and daily function outcomes

Mental health severity was assessed using 55-items including locally adapted versions of the Hopkins Symptom Checklist-25 (HSCL-25) to assess depression (15 items) and anxiety symptoms (10 items), the Harvard Trauma Questionnaire (HTQ) to assess symptoms of posttraumatic stress (16 items), and 14 additional symptoms identified during an initial qualitative study. Respondents were asked to report how frequently they experienced each symptom over the prior 2 weeks using a four-point Likert scale (0 = not at all, 1 = a little bit, 2 = a moderate amount, 3 = all the time). Average total mental health severity scores were generated using all 55 items; scores ranged from 0 to 3, with higher scores indicating greater severity. Subscales for depressive, anxiety, and posttraumatic stress symptoms, and symptoms generated from the qualitative study were also analyzed and are presented in the online Supplementary Appendix.

Daily functioning was assessed using 20 tasks of daily living reported in a prior qualitative study as relevant to local women. Women were asked to report how much difficulty they had engaging in each task over the prior 2 weeks, with responses ranging from 0 (no difficulty) to 4 (so much difficulty cannot do it). Average scores were generated; scores ranged from 0 to 4, with higher scores indicating greater functional impairment.

### Data collection

Data were collected at baseline and 2 months after the VSLA program cycle was complete in June 2012. These data were used to assess the primary outcome of intervention effectiveness. To explore whether any effects were further maintained among VSLA participants, an additional follow up was conducted 8 months later among only the original VSLA participants. All follow-up interviews contained the same questions as the baseline. For the 2-month follow-up, interviewers were blind to study condition.

### Sample size

We conducted a pre-hoc pair-wise comparison to determine number of participants needed to find a clinically significant (0.5 points on a scale from 0 to 3) greater reduction in average symptom scores by treatment condition. The decision to use 0.5, which is a half a point more improvement among participants compared with controls, was based on wanting to find not only statistically significant differences but also clinically meaningful ones. Assuming a 20% drop-out rate, we calculated that 180 participants per study arm for 80% power to detect a design effect of 2.0 (to compensate for correlations within study arms).

### Analytic methods

Baseline variables were compared across intervention conditions using student *t* tests for continuous variables, ranksum tests for non-normally distributed variables, and Pearson's chi-squared or Fisher's exact tests for binary variables. Demographics and other factors associated with loss-to-follow up were explored using logistic regression.

Indicators of intervention effect were derived comparing intervention and control participants' mean changes across scores from baseline to 2-month follow-up assessment. Analyses were on intent-to-treat sample. Item-level missing data were imputed based on mean values for other items in the measure. For covariates, missingness in any variable under 5% was handled with list-wise deletion. Maximum likelihood estimated random effects models (Stata XTMIXED) were used for continuous outcomes, with a robust variance estimator. This longitudinal model, with three random effects (participant, VSLA group, and village) used all observations while accounting for correlations at each level. For binary outcomes, maximum likelihood estimated multilevel mixed effects logistic regression was used (Stata XTMELOGIT). In all models, fixed effects included time of assessment (baseline or 2-month follow up) and treatment condition. For the mental health and function outcomes, effect sizes, reflecting regression adjustments, were calculated using Cohen's *d*. All comparisons were planned and tests were two-sided.

Demographic and baseline characteristics that differed among the conditions were included as control variables in all analyses. Variables related to loss-to-follow up were used to predict follow-up probability, which was then used to generate a weighting variable; all analyses included the inverse probability of being followed up. Separate weights were generated and combined for the probability of being followed up at post intervention and at 8 month follow up for the VSLA participants. A *p* value of <0.05 was considered statistically significant.

## Results

**Fig. 1.**
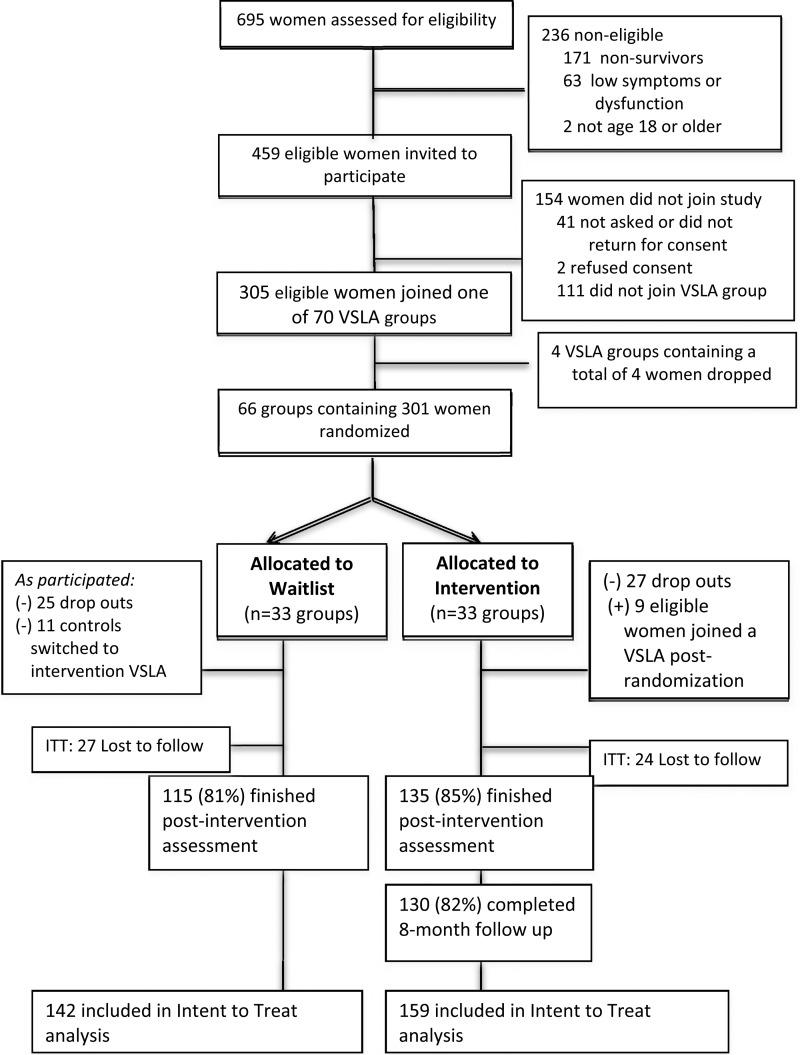
Flow chart of study participants.

A total of 695 women provided informed consent and were screened for eligibility (Fig. 1). Of these, 459 (66%) met inclusion criteria and 301 (66%) agreed to participate and joined a VSLA group. At post-intervention follow-up, 52 women (17%) were unable to be re-assessed, 28 (20%) controls and 24 (15%) VSLA participants. At the 8-month follow-up of VSLA participants, 141 (89%) were assessed. Variables associated with loss-to-follow up post intervention included: village (three study villages had security issues); lived in territory of origin (i.e. women living in their territory of origin were more likely followed up); length of time in current village (i.e. women who had lived in their villages longer were more likely followed up); language spoken (Swahili speakers were less likely followed up); and among VSLA women, total number of people living in the household and baseline total mental health symptom score. For women in VSLA groups, 96% of the study participants completed the VSLA program, defined as being a member at the end of the cycle.

Demographic information about VSLA and control participants is presented in Table 1. Table 2 presents baseline economic, social and mental health outcomes by intervention arm. Greater than two-thirds of women were living in the same area where they were born. Mean participant age was early 40s, and on average, women reported having received approximately 2 years of formal education. On most variables, women in the study conditions were similar at baseline, though women in the control arm reported experiencing and witnessing more types of traumatic events and were slightly more ethnically diverse. Women in both arms were similar in baseline scores on study outcomes; however, control women worked statistically significantly more total economic hours in the past week, had more people they could rely on for assistance, expressed greater group membership, and spent more money on food *per capita* in the household. Review of the monitoring logs of the VSLA groups identified that across the VSLA cycle, the average VSLA participant saved US$36.35 and received an average share-out return of US$51.95, representing an average profit of 42.4%.

**Table 1.** Study sample characteristics at trial baseline *(*n = *301)*

Variable	VSLA (*n* = 159)		Control (*n* = 142)	
	Mean or no.	Range	Mean or no.	Range
Demographic characteristics				
Age in years, Mean (s.d.)	40.1 (11.7)	18–77	41.5 (12.8)	20–70
Years of education completed, Mean (s.d.)	2.0 (3.1)	0–12	1.9 (3.0)	0–12
Number of people living in home, Mean (s.d.)	7.5 (2.8)	1–15	7.4 (3.1)	1–22
Number of children responsible for, Mean (s.d.)	4.5 (2.5)	0–16	4.5 (2.5)	0–17
Marital status, No. (%)				
Single/divorced	8 (5.1%)		2 (1.4%)	
Married	86 (54.1%)		76 (53.5%)	
Separated	32 (20.1%)		31 (21.8%)	
Widowed	33 (20.8%)		33 (23.2%)	
Living in territory of origin, No. (%)	111 (70.0%)		105 (73.9%)	
Currently participating in a loan/grant activity, No. (%)	24 (15.1%)		18 (12.7%)	
Ethnicity, No. (%)				
Mushi	135 (84.9%)		117 (82.4%)	
Mufuliru	19 (12.0%)		12 (8.5%)	
Other	5 (3.1%)		13 (9.2%)	
Trauma exposure				
Personally experienced sexual violence, No. (%)	87 (54.7%)		97 (68.3%)	
Personally witnessed sexual violence, No. (%)	140 (88.1%)		122 (85.9%)

**Table 2.** Study outcome measures at trial baseline *(*n = *301)*

Variable	VSLA (*n* = 159)		Control (*n* = 142)	
	Mean or no.	Range	Mean or no.	Range
Economic functioning				
Total economic hours worked in past 7 days, Mean (s.d.)^a^*	34.8 (21.7)	0–85	41.6 (23.7)	0–90
Total paid hours worked in past 7 days, Mean (s.d.)^b^	18.1 (16.5)	0–73	22.5 (19.9)	0–84
Food expenditure *per capita* in CDF, Mean (s.d.)*	874.9 (871.2)	0–5032	1188.5 (996.0)	0–4820
Number of breeding animals owned, Mean (s.d.)	3.8 (6.3)	0–45	4.1 (6.9)	0–63
Social functioning				
Access to social resources, Mean (s.d.)*	1.77 (2.9)	0–16	2.28 (2.9)	0–16.5
Group membership index, Mean (s.d.)*	3.45 (1.7)	0–8	3.89 (1.9)	0–8
Stigma score, Mean (s.d.)	1.42 (0.82)	0.1–3	1.54 (0.77)	0–3
Mental health symptom and daily functioning				
Average total symptom score, Mean (s.d.)	2.0 (0.5)	1.0–2.9	2.0 (0.5)	1.0–2.9
Average functional impairment score, Mean (s.d.)	1.8 (0.7)	0.6–3.8	1.7 (0.7)	0.5–3.2

a Sample includes *n* = 155 in VSLA and *n* = 140 in control because of issues with missingness.

b Sample includes *n* = 158 in VSLA and *n* = 140 in control because of issues with missingness.

* Between arm differences significant at *p* = 0.05 level.

Table 3 presents results for intervention effects on study outcomes. Two months after the intervention, *per capita* food consumption was on average 25% greater among the VSLA participants compared with controls (*p* = 0.010). On average, VSLA participants reported having approximately 1.5 more animals for breeding than controls, but this difference was only marginally significant (*p* = 0.078). While both treatment and control groups experienced an average decline in paid hours worked between baseline and follow up, VSLA participants experienced a marginally significant smaller reduction (*p* = 0.053). There was also a smaller decrease in total economic hours worked among VSLA participants as compared with controls, but this difference was not statistically significant (*p* = 0.190). Women in VSLA groups reported on average more than 10% greater reduction in experience of stigma (*p* = 0.038). There were no significant differences between the VSLA and control participants in degree of community participation or access to social resources. Changes in mental health severity and daily functioning scores were not statistically different between VSLA and control participants.

**Table 3.** Effect of VSLA program on mental health, functioning, economic and social outcomes *(*n = *283)*^a^

	Observed		% Change from baseline		Effect estimate*	
	VSLA	Control	VSLA	Control	Beta, *p* value (95% CI)	Cohen's *D*
Economic outcomes						
Hours paid work (last 7 days)^b^						
Baseline, Mean (s.d.)	18.10 (16.53)	22.53 (19.87)	−18%	−31%	0.17, *p* = 0.053 (−0.002 to 0.34)	
Post-Intervention, Mean (s.d.)	14.78 (14.53)	15.53 (14.84)				
Total economic hours worked- paid or unpaid (last 7 days)						
Baseline, Mean (s.d.)	34.84 (21.65)	41.60 (23.67)	−19%	−25%	3.71, *p* = 0.190 (−1.84 to 9.26)	
Post-Intervention, Mean (s.d.)	28.07 (20.92)	31.14 (19.93)				
Food consumption *per capita*^b^						
Baseline, Mean (s.d.)	875 (871)	1189 (996)	74%	47%	0.25, *p* = 0.010 (0.06–0.44)	
Post-intervention, Mean (s.d.)	1520 (1264)	1747 (1308)				
Number of animals for breeding						
Baseline, Mean (s.d.)	3.79 (6.34)	4.09 (6.92)	50%	0	1.56, *p* = 0.078 (−0.17 to 3.29)	
Post-intervention, Mean (s.d.)	5.69 (8.29)	4.09 (4.58)				
Social outcomes						
Access to social resources^b^						
Baseline, Mean (s.d.)	1.77 (2.9)	2.28 (2.9)	39%	0	0.24, *p* = 0.118 (−0.06 to 0.55)	
Post-intervention, Mean (s.d.)	2.90 (3.2)	2.29 (2.0)				
Group membership index						
Baseline, Mean (s.d.)	3.45 (1.7)	3.89 (1.9)	−9%	−5%	−0.14, *p* = 0.652 (−0.77 to 0.48)	
Post-intervention, Mean (s.d.)	3.15 (1.5)	3.68 (1.6)				
Stigma						
Baseline, Mean (s.d.)	1.42 (0.82)	1.54 (0.77)	−49%	−32%	−0.20, *p* = 0.038 (−0.40 to −0.01)	
Post-intervention, Mean (s.d.)	0.72 (0.76)	1.05 (0.86)				
Mental health and functioning						
Total symptom score						
Baseline, Mean (s.d.)	1.95 (0.51)	2.03 (0.50)	−32%	−25%	−0.10, *p* = 0.154 (−0.25 to 0.04)	0.21
Post-intervention, Mean (s.d.)	1.33 (0.66)	1.52 (0.69)				
Function score						
Baseline, Mean (s.d.)	1.76 (0.70)	1.73 (0.67)	−26%	−23%	−0.06, *p* = 0.417 (−0.20 to 0.08)	0.09
Post-intervention, Mean (s.d.)	1.30 (0.68)	1.34 (0.67)				

a This size for each analysis ranges from 240 to 295 because of log transformation for non-normal variables resulted in 0s and listwise deletion for missingness in covariates under 5%. All regressions control for baseline ethnicity, baseline number of types of traumas experienced, log baseline *per capita* food consumption and baseline total hours worked in previous 7 days.

b Outcome variable is log transformed due to non-normal distribution.

Table 4 shows changes in outcomes for VSLA participants from post-intervention to 8-month follow-up. VSLA participants reported a 20% increase in *per capita* food consumption expenditures (*p* = 0.001) and a large increase in economic hours worked in the past 7 days (*p* = 0.009). Access to social resources improved (*p* = 0.001), while group membership and experience of stigma remained stable. Total symptom severity remained stable during this time, while functional impairment declined (i.e. improved) by a small but significant amount (*p* = 0.03).

**Table 4.** Change in economic, social and mental health outcomes from post-intervention to 8 month follow up among VSLA participants *(*n = *141)*^a^

Outcome	Observed	Beta time 2–3, *p* value (95% CI)
Food consumption *per capita*^b^		
Post-intervention, Mean (s.d.)	1520 (1264)	0.21, *p* = 0.001 (0.08–0.34)
8-month follow up, Mean (s.d.)	1964 (1366)	
Hours paid work in last 7 days^b^		
Post-intervention, Mean (s.d.)	14.78 (14.53)	0.11, *p* = 0.296 (−0.10 to 0.32)
8-month follow up, Mean (s.d.)	18.77 (15.53)	
Total economic hours worked paid or unpaid in last 7 days		
Post-intervention, Mean (s.d.)	28.07 (20.92)	7.99, *p* = 0.009 (1.97–14.01)
8-month follow up, Mean (s.d.)	37.45 (23.04)	
Number of animals for breeding		
Post-intervention, Mean (s.d.)	5.69 (8.29)	−0.33, *p* = 0.608 (−1.56 to 0.91)
8-month follow up, Mean (s.d.)	5.47 (7.86)	
Access to social resources score		
Post-intervention, Mean (s.d.)	2.90 (3.2)	0.27, *p* = 0.001 (0.12–0.42)
8-month follow up, Mean (s.d.)	6.28 (11.9)	
Group membership index		
Post-intervention, Mean (s.d.)	3.15 (1.45)	0.17, *p* = 0.562 (−0.40 to 0.73)
8-month follow up, Mean (s.d.)	3.39 (1.62)	
Stigma score		
Post-intervention, Mean (s.d.)	0.72 (0.86)	0.12, *p* = 0.172 (−0.05 to 0.29)
8-month follow up, Mean (s.d.)	0.82 (0.71)	
Total symptom score		
Post-intervention, Mean (s.d.)	1.33 (0.66)	−0.02, *p* = 0.807 (−0.16 to 0.12)
8-month follow up, Mean (s.d.)	1.29 (0.67)	
Functional impairment score		
Post-intervention, Mean (s.d.)	1.30 (0.68)	−0.20, *p* = 0.033 (−0.39 to −0.02)
8-month follow up, Mean (s.d.)	1.11 (0.63)	

a All regressions control for baseline ethnicity, baseline number of types of traumas experienced, log baseline per capita food consumption and baseline total hours worked in previous 7 days.

b Outcome variable is log transformed due to non-normal distribution.

## Discussion

Female survivors and witnesses of sexual violence with elevated symptoms of mental distress were successfully integrated into a community-based economic program in eastern DRC. Compared with a control condition, VSLA participants experienced significant increases in *per capita* food expenditure and marginally significant differences in hours of paid work per week. The food expenditures outcome continued to improve among VSLA participants at the 8-month follow up. Interpretation of the number of paid hours worked was less clear, as on average, study participants in both study arms reported fewer hours of paid work per week at follow up compared with baseline; this may have been due to environmental factors such as weather (baseline and follow-up were assessed in different seasons) and/or recent violence limiting work in the prior week. While trends were in the expected direction, we did not find significant impact of VSLA participation on the other economic indicators of total hours worked or number of breeding animals owned. This is consistent with the variation seen in other VSLA research. Ksoll *et al.* (2013) found some evidence of improvements in consumption in Malawi but limited evidence of increased small business activities. In Burundi, Annan *et al*. (2013) found impacts among savings groups after the end of one cycle, including higher consumption and assets. A review by Karlan *et al.* (2012) evaluating the CARE VSLA model in Uganda, Malawi and Ghana, found some evidence of women investing more in small enterprises and improvements in food security but no change in assets after 2 years of the program.

Detailed discussion with field staff and a brief post-intervention qualitative study with VSLA participants provided some context-specific explanations about the lack of significant impact of VSLA participation on total hours of economic work. For example, women reported using VSLA funds to purchase land for cultivation, but may not have started cultivating it. Funds were also shared with other family members who used it for economic benefit not represented by the woman's amount of work. Finally, after share-out, VSLA participants reported that some savings were used to cover daily costs, including school fees, which would not be reported as economic work though clearly benefited the woman and her family.

In terms of social well-being, we saw that VSLA participants on average, compared with controls, experienced a reduction in their personal feelings of stigma. This may have been a result of incorporating the study women (sexual violence survivors) into a general population program. Alternatively, acceptance into a group may have provided confidence and increased positive interactions with others.

Despite some economic and social improvements, the VSLA groups did not impact participants' mental health. Participant symptoms declined in both the VSLA and the control groups; with slightly more but not significant reduction among VSLA participants. Among the VSLA participants, at the 8-month follow up there was little additional change in average symptom severity. This is in contrast to the effect of cognitive processing therapy with a similar population of women in DRC, where the effects of the therapy were strong and sustained, with a >50% reduction in symptoms more than 6 months after treatment completion (Bass *et al.* 2013). Our initial hypotheses as to how the VSLA program might impact mental health were based on the assumption that improved access to financial resources would reduce poverty-related stress and participation in a group economic intervention would improve self-efficacy, both outcomes that are often related to mental health. Similar to what was found in the study of a microenterprise program in Northern Uganda (Green *et al.* 2016). It may be that in this conflict-affected context mental health problems are less related to poverty and self-efficacy and more a result of ongoing instability and the endemic poverty that a small economic program cannot alleviate. Given that both groups of women, those in the VSLA program and those in the wait-control condition, showed improvement in their mental health symptoms over time, it is also quite possible that secular trends may have washed out improvements that we may have seen in a context that was more stable.

These results are in contrast to two studies showing significant improvement in economic outcomes and concurrent reductions in depressive symptoms from cash transfer programs (Ozer *et al.* 2011; Haushofer & Shapiro, 2013). Cash transfer programs by design provide a one-time sum of funds, which can in some cases be quite large, for example Haushofer & Shapiro's (2013) study in Kenya in which households received unconditional cash transfers of US$40, $400, or $1500. The cash transfer model provides a rapid influx of funds that the family can utilize as needed, including for costs associated with daily living as well as building new opportunities for income generation. In contrast, VSLA programs rely on funds already available within the community, and specifically made available by the participants themselves. Thus, the funds that women are able to earn in interest from their savings over the course of any given VSLA cycle are relatively small in comparison with cash transfers. The theory of the VSLA model is that over multiple cycles, a VSLA will build group equity and provide a sustainable, ongoing availability of loans for a wide range of participants. In our study, the average savings over the course of the full 10-month VSLA cycle approached US$40, with an average increase of approximately 40% (i.e. approximately US$16) profit at the time of payout. This is significantly lower than the lower end of the cash-transfer programs, which may help to explain some of the differences in study findings. Given that our evaluation focused on the impact of a single VSLA cycle, it may be that the delivery of this type of economic intervention requires additional ongoing dosage or must meet a specific threshold in order to realize mental health benefits and one cycle of VSLA was insufficient for survivors of sexual violence in a conflict-affected setting.

### Limitations

The principle of self-selection is crucial for maintaining trust within VSLA groups. A study challenge was ensuring enough research-eligible women actually self-selected into VSLA groups to have a large enough study samples. Fewer research eligible women ended up self-selecting per group than originally estimated, resulting in more groups needing to be included in the study.

Another limitation was related to attrition, particularly of control participants who were difficult to locate after a year. Related, of the 301 eligible women included in the intent-to-treat analysis, 11 switched from control to intervention VSLAs, 27 dropped out of their VSLA group, and 26 dropped out of their control group. A per protocol analysis was completed and results were not meaningfully different. Finally, due to the limited number of data collection time points, we are unable to quantitatively evaluate, through mediation analyses, our initial hypotheses as to the mechanism by which the VSLA program could have improved mental health outcomes.

## Conclusions

This study rigorously evaluated the impact of a group savings program for female sexual violence survivors in a conflict setting on a range of economic, social and psychological outcomes. We learned that it is possible to implement targeted strategies to include sexual violence survivors in VSLA groups in a safe and ethical manner, and that sexual violence survivors even those with severe trauma symptoms, can benefit from this participation. Participation data indicated that study women participated as frequently in meetings and payment/loan activities as non-study women. In terms of improving economic, social and psychological well-being of female survivors of sexual violence, this study shows impacts for some social and economic outcomes, but no psychological improvements, suggesting that more targeted mental health interventions may be needed to improve their psychological well-being.

## Supplementary material

For supplementary material accompanying this paper visit http://dx.doi.org/10.1017/gmh.2016.13. click here to view supplementary material
