# Supplementary material for: Economic, social and mental health impacts of an economic intervention for female sexual violence survivors in Eastern Democratic Republic of Congo
Source: Glob Ment Health (Camb). 2016 Jun 6;3:e19. doi: 10.1017/gmh.2016.13 (PMC5314746; doi:10.1017/gmh.2016.13)
Supplement: Supplementary file 1 [file S2054425116000133sup.zip › S2054425116000133sup002.docx]

| **Appendix:** Additional outcomes evaluated of VSLA intervention compared with control condition | | | | | | | | | | | | |
| --- | --- | --- | --- | --- | --- | --- | --- | --- | --- | --- | --- | --- |
|  | **Observed** | | | | **% Change from baseline** | | | | **Effect Estimate *** | | | |
|  | **VSLA** | | **Control** | | **VSLA** | | **Control** | | **Beta, p-value**  **(95% CI)** | | **Cohen’s D** | |
| **Additional Economic Outcomes** |  | |  | |  | |  | |  | |  | |
| Unpaid total hours worked (last 7 days)  Baseline, Mean (SD)  Post-Intervention, Mean (SD) | 53.49 (19.36)  51.95 (23.63) | | 57.12 (19.35)  55.88 (17.48) | | -3% | | 2% | | -0.98, p=0.709  (-6.10, 4.15) | |  | |
| Unpaid economic hours worked (last 7 days) ^1^  Baseline, Mean (SD)  Post-Intervention, Mean (SD) | 21.19 (18.58)  16.32 (15.75) | | 23.54 (19.35)  17.59 (15.99) | | -23% | | -25% | | 0.15, p=0.378  (-0.19, 0.50) | |  | |
| Unpaid domestic hours worked (last 7 days)  Baseline, Mean (SD)  Post-Intervention, Mean (SD) | 36.81 (17.62)  37.06 (19.26) | | 37.90 (18.65)  41.26 (17.65) | | 1% | | 9% | | -3.92, p=0.195  (-9.86, 2.01) | |  | |
| Total hours worked (last 7 days)  Baseline, Mean (SD)  Post-Intervention, Mean (SD) | 70.47 (24.09)  65.81 (29.68) | | 77.13 (24.52)  70.16 (22.20) | | -7% | | -9% | | 2.00 p=0.474  (-3.48, 7.48) | |  | |
| Any paid work (last 7 days)  Baseline, N (%)  Post-Intervention, N (%) | 125 (79%)  98 (73%) | | 120 (86%)  90 (78%) | | -6% | | -8% | | 0.23, p=0.639  (-0.73, 1.19) | |  | |
| Asset Index Factor Score  Baseline, Mean (SD)  Post-Intervention, Mean (SD) | -0.07 (0.97)  -0.06 (0.96) | | 0.08 (1.03)  0.07 (1.04) | | 14% | | -13% | | 0.06, p=0.524  (-0.12, 0.23) | |  | |
| **Additional Social Functioning Outcomes** |  | |  | |  | |  | |  | |  | |
| Social coping score  Baseline, Mean (SD)  Post-Intervention, Mean (SD) | | 2.05 (0.73)  2.13 (0.66) | | 2.15 (0.67)  2.16 (0.66) | | 4% | | 0% | | 0.05, p=0.716  (-0.21, 0.30) | |  |
| Community functioning score  Baseline, Mean (SD)  Post-Intervention, Mean (SD) | | 1.28 (0.97)  0.75 (0.82) | | 1.13 (0.88)  0.75 (0.83) | | 41% | | 34% | | -0.13, p=0.151  (-0.31, 0.05) | |  |
| Family functioning score  Baseline, Mean (SD)  Post-Intervention, Mean (SD) | | 1.77 (1.05)  1.31 (0.98) | | 1.76 (1.04)  1.22 (1.04) | | 26% | | 31% | | 0.13, p=0.405  (-0.17, 0.43) | |  |
| **Additional Mental Health Outcomes** | | | | | | | | | | | | |
| Depression score  Baseline, Mean (SD)  Post-Intervention, Mean (SD) | 1.89 (0.54)  1.29 (0.70) | | 1.96 (0.57)  1.45 (0.71) | | -32% | | -26% | | -0.08, p=0.212  (-0.22, 0.05) | | 0.15 | |
| Anxiety score  Baseline, Mean (SD)  Post-Intervention, Mean (SD) | 2.22 (0.61)  1.58 (0.82) | | 2.31 (0.59)  1.80 (0.82) | | -29% | | -22% | | -0.11, p=0.325  (-0.33, 0.11) | | 0.18 | |
| Trauma score  Baseline, Mean (SD)  Post-Intervention, Mean (SD) | 1.94 (0.58)  1.39 (0.74) | | 1.98 (0.58)  1.49 (0.76) | | -28% | | -25% | | -0.05, p=0.563  (-0.22, 0.12) | | 0.09 | |
| Qualitative score  Baseline, Mean (SD)  Post-Intervention, Mean (SD) | 1.95 (0.51)  1.30 (0.67) | | 2.03 (0.51)  1.52 (0.70) | | -33% | | -25% | | -0.13, p=0.087  (-0.29, 0.02) | | 0.26 | |

^1^ Outcome variable is log transformed due to non-normal distribution
