# Supplementary material for: Economic, social and mental health impacts of an economic intervention for female sexual violence survivors in Eastern Democratic Republic of Congo
Source: Glob Ment Health (Camb). 2016 Jun 6;3:e19. doi: 10.1017/gmh.2016.13 (PMC5314746; doi:10.1017/gmh.2016.13)
Supplement: Supplementary file 1 [file S2054425116000133sup.zip › S2054425116000133sup001.docx]

# Intervention: Village Savings and Loans Associations

##

## **VSLA as the social-economic intervention**

Given that pervasive sexual violence in DRC is the product of a complicated web of violent power relations and structural factors, women and girls need not only protection from violence but should also be healed and empowered to have their contributions to their families, their communities, and their society recognized. The need for economic programming to support survivors in meeting their day-to-day needs was frequently articulated by IRC’s local partners and beneficiaries. Furthermore, during the qualitative study conducted for this research project, poverty and related problems were the most frequently cited problems across all villages. Accordingly, and in line with its commitment to implement programs that are both evidence-based and evidence-generating, and based on the IRC’s experience using this model elsewhere, the Village Savings and Loans Associations (VSLAs) model was selected as an appropriate intervention for this population and was implemented to evaluate its impact on the social, psychological, and economic well-being of survivors of sexual violence. A model based on local capacity and trust among the members, the IRC identified VSLA as an important tool to provide access to financial resources, foster social cohesion and potentially promote social reintegration of survivors.

The IRC has experience with training and running VSLAs in the region, as well as completing a randomized controlled trial of the program evaluating impacts on household assets and child outcomes in Burundi. Lessons learned from the implementation of this trial were used in the planning and implementation of the trial in the DRC. The implementation of income generating activities in groups that include SV survivors is based on the idea that bringing women together to build socioeconomic efficacy can potentially counteract the loss of autonomy, self-sufficiency, and social relations often associated with sexual violence. VSLA groups made up of only survivors were not considered due to stigma concerns.

##

## **Description of VSLAs**

The Village Savings and Loans Associations (VSLA) model was developed by CARE International, based on indigenous savings and loans groups in Africa. VSLAs provide a community-managed mechanism for savings, loans and insurance for people who cannot access banks or microfinance institutions.

The standard VSLA model begins with a series of community meetings where attendees are introduced to the basic principles of a VSLA and the support a VSLA would receive from the implementing organization. Attendees are also explained the importance of a self-selected membership and are encouraged to begin forming potential VSLA groups. This was adapted slightly for the study as explained below. Once self-selected groups of 15-25 members are formed, each independent association is trained in the management of the savings and loans activities. Each member is required to save money in a common loan fund. Savings are done in the form of shares and each member can purchase 1-5 shares at each meeting. The value of a share is established by the members at the beginning of the saving cycle. Once the group as accumulated a certain level of savings, it starts to give out loans to its members. As loans are disbursed to group members, they are repaid with interest, producing a return on savings. Members must also contribute to a solidarity fund, which serves as insurance for members in the case of an emergency, such as a birth or a death in the family.

As VSLAs operate through pooling money and disbursing loans that are later repaid, individual self-selection of groups is considered by IRC as an essential component of VSLAs. Members are called to choose each other based on mutual trust, a reputation of honesty, and reliable capacity to save money regularly, even if only a small amount. Trust is also essential in the DRC context to ensure confidentiality and the group’s security. Moreover, social pressure is the only mechanism that VSLAs have to ensure members’ accountability and solvency and it needs to be built on pre-existing social bonds. VSLA is empowering not only in the way members are selected, but because participants build upon their own savings, and then following a few months of intense training and follow-up, run the associations themselves. No outside capital is ever given to the association. The IRC’s Women’s Protection and Empowerment programs work with women-only VSLAs in most sites to ensure their voices are guiding decision-making, that they build alternative social support networks and that they access economic opportunities that are often not available to them.

## **Implementation of VSLAs**

The implementation of VSLAs occur in three phases (43). Movement from one phase to the next depends on the groups’ readiness as assessed by the IRC staff and supervisors.

- **Phase 1: The preparatory phase** – IRC staff are trained on implementation of VSLAs using a participatory training curriculum designed to ensure participants acquire both knowledge and skills to train, monitor and coach VSLA groups for success. The training also includes building the capacity of staff to monitor individual group level information and overall program performance. In addition, during this phase community meetings and meetings with community leaders are held to introduce VSLA to communities and mobilize the self-selection and formation of VSLA groups. Meetings with potential VSLA groups are held to set clear expectations about the program and make sure that potential members understand the requirement and commitment expected of them.
- **Phase 2: The intensive training phase** - The intensive phase is a period of three to four months during which VSLA groups are trained on how to manage VSLA activities, with frequent (normally weekly) supervision from IRC staff. Training takes place during weekly meetings and comprises of six modules. The first five trainings happen over five consecutive weeks. Module 6 takes place one month after module 5. During this phase, group members begin saving, taking loans and repaying loans.
- **Phase 3: The supervision phase** - Three to four weeks after the groups complete Module 6 training, they transition into the supervision phase, which normally lasts about 8 months. During this period, VSLA groups conduct their weekly activities on their own, with less frequent guidance and support from IRC staff. The purpose of the supervision phase is to support VSLA groups to become confident in managing their activities, monitor group progress, provide additional training when necessary, and support the group in finding their own solutions to problems. The supervision phase is divided into two stages, the development and maturity phases:
  - The development phase lasts for four to five months after groups finish their training. In this phase, members start to take full responsibility for running meetings. IRC staff visits each group every 1-2 weeks, depending on need. During each visit, IRC staff pays close attention to how well VSLA procedures are being respected and, if necessary, will review basic principles and refresh some of the training modules. Overall, the purpose of this phase is to help the group become confident in its capacity to manage its operations.
  - The maturity phase lasts three to four months after the end of the development phase and involves three key visits. Two of these are supervision visits, to ensure that the group can function without any outside help and to begin preparing for sharing out of the group’s pooled savings. If the group needs additional training or supervision, the cycle can be extended. If the association is ready to be independent, the community workers make a third visit on the last meeting of the cycle to facilitate the share-out process.

Once the first cycle of savings and loan activities and share out is complete, the VSLA groups are free to decide whether or not to continue for a second cycle. Since these groups are now considered autonomous and independent, the IRC would only provide them with support sporadically or should a major concern arise.

IRC has implemented VSLAs in several countries and has found the results promising. IRC first implemented VSLA activities as part of their Women’s Protection and Empowerment program in Burundi and has since expanded them to many other countries including the DRC, Ivory Coast, Sierra Leone, Liberia, Uganda, and Haiti. However, the program in DRC was the first time IRC adapted the VSLAs to target SV survivors.
